# Clinical, electrophysiologic, and histopathologic profile, and outcome in idiopathic inflammatory myositis: An analysis of 68 cases

**DOI:** 10.4103/0972-2327.74190

**Published:** 2010

**Authors:** K. N. Ramesha, Abraham Kuruvilla, P. S. Sarma, V. V. Radhakrishnan

**Affiliations:** Department of Neurology, Sree Chitra Tirunal Institute for Medical Sciences and Technology, Trivandrum, India; 1 Department of Biostatistics, Sree Chitra Tirunal Institute for Medical Sciences and Technology, Trivandrum, India; 2 Department of Neuropathology, Sree Chitra Tirunal Institute for Medical Sciences and Technology, Trivandrum, India

**Keywords:** Final outcome, myositis, prognosis, survival rate

## Abstract

**Objectives:**

To study the electroclinical and histopathologic profile of idiopathic inflammatory myositis (IIM) with reference to prognosis and survival rate.

**Materials and Methods:**

Diagnosis of IIM was based on the Bohan and Peter criteria. Patients who improved and those whose condition worsened or who expired due to IIM per se at last follow-up were classified to have favorable and poor outcomes, respectively. Fisher’s exact test was used for univariate analysis of prognostic factors.

**Results:**

The study cohort consisted of consecutive 68 patients with IIM. The mean age at diagnosis was 36.5 years and females constituted 71%. Of these patients, 62% had definite IIM, 49% had polymyositis, 20% had dermatomyositis, and 29% had overlap syndrome. The mean follow-up period was 5.4 years. Prednisolone alone was used in 55 (80%), and azathioprine (1–3 mg/kg/day) alone in 12 (17.6%) as the initial treatment. Relapse of IIM with drug withdrawal was seen in 15 patients (22%); 70% had favorable outcome and 16% had expired. The treatment delay of ≤6 months (*P* = 0.001), absence of cardiac or lung involvement (*P* < 0.001), and positive biopsy (*P* = 0.033) were predictive of a favorable prognosis in the univariate analysis. In multivariate analysis, only the duration of illness of ≤6 months (*P* = 0.008) and the absence of cardiac or lung involvement (*P* = 0.001) predicted the favorable outcome at last follow-up. Cumulative survival rate was 95% at 1 year, 86% at the 5th year, and 80% at the 10th year.

**Conclusions:**

Approximately, two-thirds of the patients showed good electroclinical and histopathologic correlations and an equal number improved with treatment. The treatment delay (≥6 months), presence of cardiac or pulmonary involvements, and negative muscle biopsy are bad prognostic factors.

## Introduction

The idiopathic inflammatory myositis (IIM) is a rare heterogenous group of disorders in which the primary pathologic process is inflammation within the muscle.[1,2] The disease has been divided into dermatomyositis (DM), polymyositis (PM), IIM associated with other connective tissue disorders (overlap syndromes), neoplasia-associated IIM, and inclusion body myositis (IBM).[1,3] The diagnosis and treatment are straightforward and rewarding in a majority of the cases.[3,4] The treatment is based both on expert opinion[3,4] and systematic analysis of a few randomized controlled trials.[5] Mortality is mainly because of other organ involvement or due to underlying malignancy.[6,7] The outcome and prognostic factors in IIM vary widely in the literature.[1–4,6–10] In this background, we aimed to evaluate the clinical, electrophysiologic, and histopathologic profile and their correlations in IIM and also to estimate the overall response rates to standard treatment and relapse rate with drug tapering, to assess the potential prognostic factors and to calculate the mortality rates and their causes in the study cohort.

## Materials and Methods

All the consecutive cases of IIM treated in the Department of Neurology at Sree Chitra Tirunal Institute for Medical Sciences and Technology, Trivandrum, India, from 1990 to April 2004 were reviewed from the medical records. The patients with a discharge diagnosis of IIM from April 2004 to December 2006 were prospectively followed-up for a minimum period of 2 years. A systematic chart review was done to collect the demographic data, clinical features, and investigations, including serum enzyme assay, electromyography (EMG) and muscle biopsy findings, treatment details, and complications related to treatment. The patients were followed-up initially at 3-month intervals till the end of the 1st year after the diagnosis, then yearly till last follow-up wherever possible. Patients who did not come for follow-up were contacted either by letter or by telephone regarding their treatment and health status at last follow-up. During each follow-up, clinical features, serum creatine phosphokinase (CPK) value and erythrocyte sedimentation rate (ESR) were noted along with the response to treatment.

The diagnosis of IIM was made according to diagnostic criteria proposed by Bohan and Peter.[1] They were subclassified into PM, DM, IBM, overlap syndrome with other connective tissue disorders and neoplasia-associated IIM. Patients with symptoms for less than 3 months duration at presentation were classified as having acute IIM. Grading of muscle weakness was done according to the standard MRC grading system. For prognostication, a muscle power of 4/5 and ≤3/5 at hip girdle and shoulder region were taken as mild and severe weakness, respectively.

The outcome at last follow-up was divided into excellent, favorable, and poor outcome. One who continued to be in complete remission after stopping the treatment was classified to have excellent outcome. Those who improved from the baseline neurologic status or became asymptomatic were classified to have favorable outcome. Those who expired due to IIM per se and those who worsened or did not improve with treatment were classified to have poor outcome. EMG was done by experts in the interpretation of EMG. Histopathology of the muscle was interpreted by expert neuropathologist, and hematoxylin and eosin stain, modified Gomori’s trichrome stain, and periodic acid Schiff (PAS) were used in all patients. Dystrophinopathies were ruled out by appropriate immunohistochemistry in patients with atypical clinical features.

For statistical analysis, Fisher’s exact test was used for the univariate analysis of various prognostic factors. The factors that were found either showing a positive trend or having a statistically significant bearing on the outcome were then subjected to multiple logistic regression analysis. Cumulative survival rate at each year up to last follow-up were calculated by Kaplan–Meier method. The *P* value≤ 0.05 was considered as statistically significant.

## Results

A total of 85 cases were collected in the retrospective group from 1990 to 2004, but six of them were excluded from the study as biopsy and EMG study unequivocally showed an alternate diagnosis. The prospective group included nine cases, which were recruited from April 2004 to December 2006. Patients with IBM (n=3), children below the age of 16 (n=12), and those with less than 2 years of follow-up (n=5) were excluded from the analysis. Hence, study cohort totally included 68 cases of IIM.

### Clinical features

The mean age at presentation was 36.5 years (range, 16–68 years), 13% of the patients were older than 50 years, 72% were females, 25% had presentation less than 3 months after the onset of symptoms, and 50% patients had muscle pain. Half of them had tenderness on examination, 30% and 66% had severe and mild weakness in the shoulder girdle, respectively, whereas 35.2% and 62.4% had severe and mild weakness in the hip girdle, respectively. Two patients had upper girdle onset and 38% were not ambulant without support at the time of presentation. All had symmetrical weakness. Fasciculation was noted in only three patients. A significant muscle wasting was seen in 28.5% patients and all of them were symptomatic for more than 6 months prior to presentation. Seventy-six percent of the patients had neck muscle weakness at the time of initial evaluation. The neck flexion was more affected than neck extension. Thirty-four percent of the patients had bulbar muscle weakness in the form of dysphagia and nasal regurgitation. More than half of the patients with dysphagia had DM. A mild degree of bifacial weakness was seen in 20%. Deep tendon reflexes were brisk in 10%, sluggish 37%, and were absent in 15%.

### Investigations

Anemia and leukocytosis were seen in 9.5% and 33% cases, respectively. ESR was raised in 38%. Serum enzymes (SGOT and SGPT) were raised in 62% and serum CPK was high in 85%, normal in 14.8%, and more than 1000 IU in 51% of the cases. Mean CPK was 3200 IU/L in the cases with value more than 1000 IU/L. Highest CPK was 25,000 IU/L. Antinuclear antibody was positive in 23 out of the total 36 patients in whom it was tested (63.9%).

EMG findings were suggestive of myopathy in 59 cases (87%). Of the 59 cases, 40 cases had classical inflammatory myositis (59%) in the form of insertional irritability, fibrillations, positive sharp waves, and myopathic motor units. Yield of paraspinal EMG was very low (6%). EMG was inconclusive or normal in two, and showed changes of isolated neurogenic etiology in six cases (6.8%). Biopsy was suggestive of inflammatory muscle disease in 51 (75%) and normal or nonspecific in 14.8%. Classical perifascicular atrophy was seen in less than 50% of the cases with DM.

### Final diagnosis at last follow-up

The mean and median follow-up period was 5.4 ± 4.0 years and 4.6 years, respectively (range 2–17 years). Forty patients had more than 5 years of follow-up. Definite IIM was seen in 62% of cases, probable IIM in 23% cases, and possible IIM in remaining 15% cases. The majority of patients were PM 33 (49%) followed by overlap syndrome 20 (29%), and DM 14 (20%).

In the DM group, 80% had classical cutaneous manifestations, such as Gottron’s scaly rashes and heliotrope eyelid rashes. In the remaining 20%, diagnosis was contributed by histopathology. In the category of overlap syndrome, out of 20 cases, seven had rheumatoid arthritis, six had systemic lupus erythematosis, and three each had systemic sclerosis and multiple connective tissue disorder. Three had calcinosis cutis. There was only one patient with IIM associated with malignancy. He succumbed to underlying carcinoma of lung with superior vena caval obstruction even though he made significant improvement in muscle power with treatment.

### Treatment

Prednisolone alone was used in 55 (80%) at 0.75–1 mg/kg/day in single daily dose), azathioprine (1–3 mg/kg/day) alone in 12 (18%), and combination of azathioprine and steroids was used as an initial treatment in one. Azathioprine was used in total 19 patients during the course of the treatment. Seven patients received azathioprine during the course of illness due to poor response to steroid or due to steroid-induced complications. In three patients, it was used in view of relapse as soon as steroids were tapered off. Steroids were tapered by the end of 3 and 6 months in 57% and 32% patients, respectively. A mean treatment duration was 2 years 6 months. Out of six patients who had respiratory compromise, intravenous immunoglobulin, and plasma exchange were used in two patients and intravenous methylprednisolone was used in four patients. Only two patients survived in this group. Methotrexate was used in three patients during follow-up. It was used as an adjunctive in one. In other two patients, it was introduced either due to steroid unresponsiveness or due to adverse effects of azathioprine and steroid. Two of them showed good improvement and one of them showed deterioration in muscle power [Table 1].

**Table 1:** Comprehensive coverage of clinical features and investigations of study cohort of 68 cases

Variables	Numbers (%)
Mean age at diagnosis	36.5 (range 16–68) years
Age more than 50 years	9 (13.2)
Male:Female ratio	1:2.4
Acute IIM	17 (25.0)
Muscle pain	34 (50.0)
Dependent for ambulation	26 (38.2)
Neck muscle weakness	52 (76.4)
Bulbar weakness	23 (33.8)
Trunk weakness	50 (73.5)
Brisk DTR	7 (10.2)
Sluggish or absent DTR	36 (52.9)
Leukocytosis	23 (33.8)
Raised ESR	26 (38.2)
Raised SGOT/SGPT	42 (61.7)
Raised CPK	58 (85.2)
EMG suggestive of myopathy	59 (86.7)
Classical EMG of IIM	40 (58.8)
Biopsy suggestive of IIM	51 (75.0)
Initial treatment: prednisolone alone	55 (80.8)
Initial treatment: azathioprine alone	12 (17.6)

IIM, idiopathic inflammatory myositis; DTR, deep tendon reflex; ESR, erythrocyte sedimentation rate; SGOT/SGPT, serum hepatic enzymes; CPK, serum creatine phosphokinase; EMG, needle electromyography.

### Complications related to drugs

Azathioprine-induced hepatitis was observed in three patients (15.8%) and leukopenia in two patients (10.5%). Both improved with discontinuation of the drug. Steroid-induced necrosis of bilateral head of femur was seen in two patients (3.6%). One patient improved with surgical replacement of head of femur. Steroid-induced transient systemic hypertension and diabetes mellitus were seen in 18 patients (27.0%). Two patients had steroid-induced psychosis.

### Analysis of final outcome

Forty-eight (70%) had favorable outcome and 39 (57%) had excellent outcome at last follow-up. During follow-up, CPK started improving along with clinical improvement in most of the patients (38 out of 46 patients, 82.6%). Relapse rate was seen in 15 patients during the follow-up (22%) and 13 of them had short-term relapse (86%).

### Cause of death

Eleven patients expired out of 68 cases (16%). Cause of death was directly related to illness in ten patients. The etiologies of death were multifactorial in four (aspiration pneumonia and septicemia related to bulbar weakness and cardiac arrhythmia), cardiac arrhythmia alone in two, and interstitial lung disease in four patients. Remaining one patient had cancer-associated delayed death. None died due to the complications of treatment. Mortality rate was same in PM and DM group. Total 13 had involvement of either myocardium or lung (15%), of which only three improved.

### Prognostic factors

There was statistically significant correlation between duration of illness before presentation, cardiac or lung involvement, and positive biopsy with the outcome at last follow-up in the univariate analysis [Table 2]. Duration of illness less than 6 months (86% vs 14%, *P* = 0.001), absence of cardiac or lung involvement (23.1% vs 76%, *P* ≤ 0.001), and positive biopsy (78% vs 21%, *P* = 0.033) had favorable outcome. There was positive trend for favorable outcome with early age of onset (less than 50 years) compared with those after the age of 50 years (76% vs 24%, *P* = 0.065). Other factors including various clinical variables, such as sex, presence or absence of neck or bulbar muscle involvement, extent of muscle weakness, EMG findings, serum CPK level, type of treatment received, and subclassification in the diagnosis did not have statistically significant correlation with the outcome at last follow-up. A multivariate logistic regression analysis was carried out on the factors that were found significant in the univariate analysis. In the multivariate analysis, only duration of illness less than 6 months (*P* = 0.008) and the absence of cardiac and lung involvement (*P* = 0.001) were found to be significant in predicting the favorable outcome [Table 3].

**Table 2:** Prognostic factors analyzed and their statistical significance at last follow-up

Variables	Factors	Favorable outcome (%)	Unfavorable outcome (%)	*P* value
Age (years)	< 50	44 (75.9)	14 (24.1)	0.065
	>50	5 (50)	4 (50)	
Sex	Males	15 (78.9)	4 (21.1)	0.302
	Females	33 (68.8)	15 (31.3)	
Duration of weakness (months)	<6	37 (86.0)	6 (14.0)	0.001
	>6	13 (54.2)	11 (45.8)	
Muscle pain	Absent	26 (76.5)	8 (23.5)	0.268
	Present	22 (66.7)	11 (33.3)	
Ambulatory without support	Yes	31 (75.6)	10 (24.4)	0.413
	No	17 (65.4)	9 (34.6)	
Neck weakness	Yes	37 (71.2)	15 (28.8)	1.000
	No	11 (73.3)	4 (26.7)	
Bulbar dysfunction	Yes	15 (65.2)	8 (34.8)	0.409
	No	33 (75.0)	11 (25.0)	
ESR	Normal	30 (75.0)	10 (25.0)	0.778
	High	18 (69.2)	8 (30.8)	
Leukocyte count	High	17 (73.9)	6 (26.1)	1.000
	Normal	31 (70.5)	13 (29.5)	
CPK	<1000 IU/mL	22 (68.8)	10 (31.3)	0.787
	>1000 IU/mL	26 (74.3)	09 (25.7)	
EMG	Positive	26 (65.0)	14 (35.0)	0.175
	Negative	22 (81.5)	5 (18.5)	
Muscle biopsy	Positive	40 (78.4)	11 (21.6)	0.033
	Negative	8 (50.0)	8 (50.0)	
Diagnosis	Polymyositis	25 (75.8)	8 (24.2)	0.486
	Dermatomyositis	9 (64.3)	5 (35.7)	
Cardiac/lung involvement	Yes	3 (23.1)	10 (76.9)	<0.001
	No	45 (83.3)	9 (16.7)	
Type of initial treatment received	Prednisolone	39 (70.9)	16 (29.1)	1.000
	Azathioprine	9 (75.0)	3 (25.0)	

ESR, erythrocyte sedimentation rate; CPK, serum creatine phosphokinase; EMG, electromyography.

**Table 3:** Results of multiple logistic regression analysis*

Variables	Adjusted odds ratio	*P* value	95%	Confidence interval
Age group ≤50 vs ≥51 years#	0.584	0.560	0.095	0.095
Duration of illness ≤6 months vs >6 months#	14.646	0.008	2.008	106.851
Biopsy negative vs positive#	0.391	0.257	0.077	1.988
Cardiac and lung involvement present vs absent#	27.964	0.001	4.004	195.276

* Four variables that were found significant in the univariate analysis were subjected to multivariate analysis;

# comparison group.

There was statistically significant positive relationship between excellent outcome when the illness was less than 3 months duration (76.2% vs 50.8%, *P* = 0.047), absence of cardiac or lung involvement (63.4% vs 23.1%, *P* = 0.013), and positive biopsy (65.1% vs 33.3%, *P* = 0.021). There was also statistically significant positive relationship between the excellent outcome at last follow-up and the muscle weakness at 3 months (69.2% vs 38.5%, *P* = 0.056) after starting the treatment.

**Figure 1:**
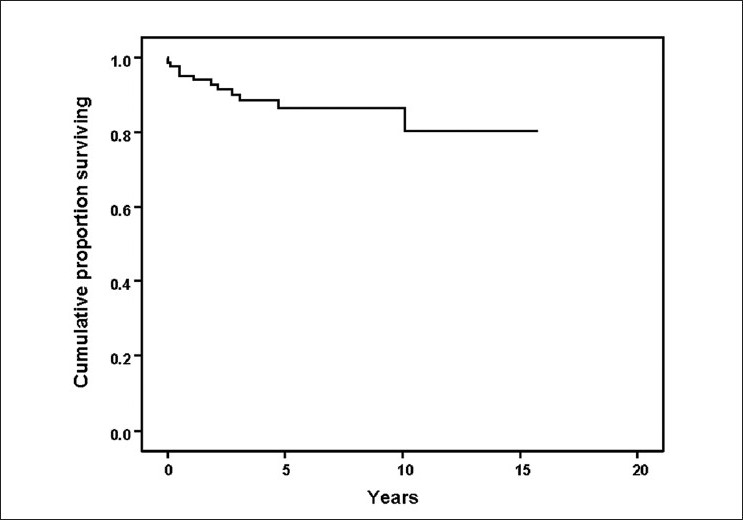
Kaplan–Meier survival analysis of 68 cases with idiopathic inflammatory myositis to depict the mortality and survival rate during the follow-up period

Cumulative survival rate was 95% at 1 year, 88% at 3 years, 86% at the end of 5 years, and 80% at 10th year by Kaplan–Meier method [Figure 1]. Survival rate was significantly better in patients without the involvement of myocardium or lung pathology (98.6%) than those with cardiac or pulmonary involvements (23.1%), *P* = 0.0000.

## Discussion

IIM is not a common neurologic illness. This study totally included 68 cases, which formed the 0.1% of 69,313 neurology outpatients and 0.6% of 14,706 neurology inpatients of this large neurology tertiary referral center in South India over 17 consecutive years. This is in agreement with the other published studies where annual incidence rate was calculated to vary between 1.9 and 7.7 per million[10,11] and prevalence rate to be 21.5, 50, and 63 per million in Canada, USA, and Japan, respectively.[10,12]

The mean age and maximum number of patients at presentation in the current study were one to two decades less than that of the majority of the published studies.[13–17] However, sex ratio was similar to other studies.[13,14,17,18] In this study, severe muscle weakness at the hip girdle and at the shoulder girdle were seen in 35% and 29%, respectively, which is in agreement with the study published by Hoffman *et al*.[19] The percentage of neck muscle involvement (76%) in the present study is much higher than in the study by Uthman *et al*.,[20] where it was seen in 23% only. One-fourth of our patients had bulbar muscle weakness at the time of initial evaluation, whereas it was only 10% in a study published by Rios *et al*.[21]

There has been a lot of variation in the prevalence of elevated CPK in IIM in various studies (64%–94%).[13,15,18,21–23] CPK was elevated in 85% of cases in the current study, which was higher than that described by DeVere *et al*.[13] who found CPK elevation in 64% of cases. We found CPK value more than 1000 IU in 50% cases, which is in agreement with that found by Uthman *et al*.[20] who found CPK > 1000 IU in 43%. The variation in CPK level among various studies may be related to the variation in the method used for analysis or due to initiation of treatment before the CPK analysis.

EMG suggestive of myopathy in IIM varies between 66% and 86%.[14,18,23,24] Myopathic features noted in our study (87%) are comparable to the aforementioned data. DeVere *et al*.,[13] Koh *et al*.,[14] and Tymms *et al*.[22] found positive biopsy in 65%, 76%, and 78% of cases, respectively. This is comparable to our data, which showed positive biopsy in 75%. The biopsy can be false negative because of patchy distribution of the pathology. Also precise diagnosis in IIM may not be easy as shown in a recent study from Mayo Clinic,[25] wherein they reviewed the clinical features, course of the disease during follow-up, and biopsy in 107 previously diagnosed cases of PM or sporadic IBM and found that 16 patients (37%) had biopsy features of PM, but clinical features of IBM. Hence they concluded that combined evaluation of biopsy and clinical features are crucial to subclassify different IIMs.

Van der Meulen[25] *et al*. concluded that pure PM entity is rare. However, in our series, 50% had PM, 30% had overlap syndrome, and 20% had DM. In our study, 75% of PM group had favorable outcome at last follow-up. This indicates that pure PM is not a very rare condition as recently thought to be. However, very high proportion of IIM being PM in this study may be because of the following reasons: some of the patients with PM with poor outcome might have had IBM as we used electron microscopy in the histopathologic diagnosis in only three patients and a repeat muscle biopsy was done in only two. None of the poor responders had a history of early falls, distal > proximal weakness, quadriceps > hip weakness, finger and wrist flexor > shoulder weakness, and selective atrophy of forearm muscles. Also, none of the patients during follow-up with PM developed other connective tissue disorders or cutaneous rashes suggestive of DM. A study from Mumbai showed that a clinical scoring system can reliably differentiate chronic IIM from the muscular dystrophies.[26]

Hoffman *et al*.[19] in a study of 27 cases with IIM found that 64% had little to no weakness within 3 months of treatment with steroids. This is comparable to our study where steroids were tapered by the end of 3 and 6 months, respectively, in 57% and 32% of our patients with clinical improvement; 57% were in remission, two-thirds had favorable outcome, and 16% expired at terminal follow-up. Similar result was noted in a study by Rios *et al*.[21] and Marie *et al*.[9] wherein remission was reported in 51% and 43%, respectively. Only 17% had full remission in another study,[27] whereas Maugars *et al*.[28] reported a favorable outcome in 84%. In a study consisting 77 cases, short-term recurrences of PM/DM (during tapering of therapy) occurred in 47% patients and long-term recurrences (after discontinuation of therapy) in 12% patients.[9] The 22% recurrence rate noted in our study is comparable to the above series.

The early treatment initiated within 6 months of onset of illness, and absence of cardiac or lung involvement had statistically significant bearing on outcome in both univariate and multivariate analyses. There was a positive trend for good prognosis with early age of onset. This is in agreement with other studies.[7,14,21,22] This is the first study that has shown positive correlation between positive biopsy and favorable prognosis. This may be because of the fact that 83% of patients who had treatment delay by less than 6 months had positive biopsy and 62% of patients who had treatment delay by more than 6 months had negative biopsy (*P* = 0.045). However, this was not found significant in multivariate analysis. Also, prognosis was excellent when improvement in muscle power was seen in the first 3 months of follow-up.

Sixteen percent mortality noted in our study was less than that reported in the majority of other studies, which ranged from 10% to 43%.[8,9,14,16,21,22,27,28] The low mortality in the current study was probably due to the fact that there were very few cases of neoplasia-associated IIM and only 13% were older than 50 years. The low rate of malignancy-associated IIM was also noted in other Indian studies.[29,30] Mortalities were mainly due to myocarditis, interstitial lung disease, and aspiration pneumonias, which are in agreement with other studies except for the proportion of cancer-related deaths.[8,23,28,31–33] There was a single mortality related to underlying malignancy in this study unlike in other studies where substantial numbers of mortalities were due to malignancy.[23,28,31–36] We did not find any difference in the death rate or statistically significant difference in the outcome between PM and DM. Some studies have shown that DM has better prognosis than PM,[34] whereas other studies have shown vice versa.[7,37]

The cumulative survival rate at 1st, 5th, and 10th year was 95%, 86%, and 80%, respectively, in the current study by Kaplan–Meier method. The survival rate in the current study was high as only one patient had malignancy and only three patients were older than 60 years. This may reflect the referral pattern. The comparison of the survival rate during follow-up between the various published studies on IIM and the current study is shown in Table 4.

**Table 4:** Comparison of the survival rate during follow-up in the various published studies on idiopathic inflammatory myositis and in the current study

Name of the study	Number of cases	1^st^ Year in %	5^th^ Year in %	10^th^ Year in %
Maugars *et al*,[28]	69	82	67	55
Miro *et al*,[34]	135	86	71	57
Sultan *et al*,[27]	46		65	53
Danko *et al*,[31]	162	95	92	92
Torres *et al*,[33]	107	92	80	71
Airio *et al*,[7]	176 PM		75	55
Airio *et al*[6]	72 DM		63	53
Current study	68	95	95	80

PM, polymyositis; DM, dermatomyositis

Limitations of the study are retrospective collection of a relatively small number of cases and a minimum follow-up period of 2 years. Electron microscopy and major histocompatibility complex detection were not used in the histopathologic diagnosis, so some DM and IBM might have been misdiagnosed to have PM. Exhaustive workup for malignancy in DM were not carried out. Hence, there is a possibility of missing some malignancy-associated IIM.

## Conclusion

Clinical, electrophysiologic, and histopathologic correlations were seen in two-thirds of the patients with IIM. More than 50% had excellent outcome and two-thirds had a favorable outcome ultimately. Majority of the relapse was due to premature withdrawal of the medications. The early commencement of the treatment, absence of cardiac or pulmonary involvement, and positive muscle biopsy findings are good prognostic factors. Patients who are likely to show excellent improvement will likely show improvement in the initial 3 months of the treatment period.
